# Immune Response and Lipid Metabolism Gene Polymorphisms Are Associated with the Risk of Obesity in Middle-Aged and Elderly Patients

**DOI:** 10.3390/jpm12020238

**Published:** 2022-02-08

**Authors:** Anastasia Ponasenko, Maxim Sinitsky, Varvara Minina, Anna Vesnina, Maria Khutornaya, Alexander Prosekov, Olga Barbarash

**Affiliations:** 1 Laboratory of Genome Medicine, Research Institute for Complex Issues of Cardiovascular Diseases, 650002 Kemerovo, Russia; sinitsky.maxim@gmail.com (M.S.); hutomv@kemcardio.ru (M.K.); 2 Department of Genetic and Fundamental Medicine, Kemerovo State University, 650000 Kemerovo, Russia; vminina@mail.ru; 3 Department of Bionanotechnology, Kemerovo State University, 650000 Kemerovo, Russia; koledockop1@mail.ru (A.V.); aprosekov@rambler.ru (A.P.); 4 Research Institute for Complex Issues of Cardiovascular Diseases, 650002 Kemerovo, Russia; barbol@kemcardio.ru

**Keywords:** genetic polymorphism, obesity, inflammation, immunity, lipid metabolism

## Abstract

More than two billion people around the world are overweight or obese. Even in apparently healthy people, obesity has a potent effect on their quality of life. Experimental data indicate the role of infectious agents in systemic inflammation, revealing a correlation between the dietary habits of people with obesity and the level of systemic inflammation mediators, serum lipid concentration, and hormonal and immune status. This study aimed to determine the association of immune response and lipid metabolism gene polymorphisms with the risk of obesity. This study included 560 Caucasian participants living in Western Siberia (Russian Federation). A total of 52 polymorphic sites in 20 genes were analyzed using the 5′ TaqMan nuclease assay. Four risk-associated polymorphic variants were discovered—two variants in immune response genes (*IL6R* rs2229238, OR = 1.92, 95% CI = 1.36–2.7, *p* = 0.0002 in the dominant model; *IL18* rs1946518, OR = 1.45, 95% CI = 1.03–2.04, *p* = 0.033 in the over-dominant model) and two variants in lipid metabolism genes (*LPA* rs10455872, OR = 1.86, 95% CI = 1.07–3.21, *p* = 0.026 in the log-additive model; *LEPR* rs1137100, OR = 2.88, 95% CI = 1.52–5.46, *p* = 0.001 in the recessive model). Thus, polymorphisms in immune response and lipid metabolism genes are potentially associated with the modification of obesity risk in the Caucasian population.

## 1. Introduction

Despite specific public health policies targeting the obesity epidemic, more than two billion people around the world are overweight or obese [1]. It is predicted that one-fifth of the working-age population will be obese by 2025; the increase in the number of obese patients is accompanied by significant socio-economic losses [2], which determine the improvement of treatment and diagnosis of this pathology, as well as the assessment of individual susceptibility to its development.

Obesity is a multifactorial disease characterized by excessive accumulation of adipose tissue, accompanied by a low-grade chronic inflammation. The triggers of this inflammation are poorly studied, but it is known that the degree of inflammation correlates with the severity of obesity-associated pathologies, which suggests that understanding the inflammatory response may improve the treatment strategies of such diseases [3,4]. In addition to inflammation, impaired lipid metabolism can be a trigger of obesity [5,6].

It is known that the activity of molecules involved in inflammation and lipid metabolism is genetically determined. Genome-wide association studies (GWAS) allow identifying genetic variants associated with susceptibility to overweight and obesity [7]. In adults, the strongest associations with the risk of obesity were discovered for single-nucleotide polymorphisms (SNPs) in the *FTO*, *MC4R*, *TMEM18*, *TNNI3K*, *SEC16B*, *GNPDA2, POMC*, *RPGRIP1L*, *IRX3*, and *IRX5* genes [8,9,10,11]. Moreover, it was shown that the genetic susceptibility to obesity is modified in a population-related manner [12,13,14]. Despite the previously obtained results, some issues related to the genetics of obesity, including the role of SNPs in genes involved in the inflammation and lipid metabolism pathways mediating obesity, are still poorly investigated, and the available results are contradictory. Therefore, understanding the role of genetic factors controlling the different pathways underlying the pathogenesis of obesity, particularly inflammation and lipid metabolism, plays a very important role in the development of personalized prevention strategies, especially in at-risk groups.

This study aimed to determine risk-associated polymorphic variants in immune response and lipid metabolism genes in obese middle-aged and elderly Caucasian patients.

## 2. Materials and Methods

### 2.1. Group Description

The present study included 560 Caucasian individuals aged 44 to 75 years (mean age of 59 years) that were long-term residents (at least three generations) in Western Siberia (Russian Federation) and undergoing a screening examination at the Research Institute for Complex Issues of Cardiovascular Diseases (Kemerovo, Russian Federation). Patients with cancer, autoimmune and mental diseases, and acute or exacerbated chronic infections associated with the inflammatory process were excluded from the study to avoid confounding effects. According to the World Health Organization age standards (2015), the patients included in this study were classified into two age groups: middle-aged (age ≤60 years) and elderly (age >60 years). 

Obesity was defined as a body mass index (BMI) of 30 kg/m^2^ or greater. In the studied group, the mean BMI was 28 kg/m^2^, ranging from 17 kg/m^2^ to 41 kg/m^2^. According to this stratification, 220 individuals (39%) were obese, and 340 individuals (61%) had a normal BMI. The complete characteristics of patients included in this study are presented in Table 1. 

The design of this study was approved by the Local Ethical Committee of the Research Institute for Complex Issues of Cardiovascular Diseases (Kemerovo, Russian Federation). All individuals included in this study provided written informed consent to participate in the examination. This study was performed in accordance with the World Medical Association Declaration of Helsinki (ethical principles for medical research involving human participants with amendments in 2000) and Good Clinical Practice.

### 2.2. Molecular Genetic Testing

Genomic DNA was isolated using the routine phenol–chloroform extraction method from whole blood collected from the cubital vein in vacuum tubes with K3EDTA and stored at −80 °C until the next stage of the experiment.

SNPs were selected according to the following criteria: (i) location within immune response and lipid metabolism genes; (ii) minor allele frequency >5% in Caucasian populations; (iii) functional consequences and related studies on their role in obesity pathogenesis. Accordingly, we selected 52 SNPs in 20 genes. The complete characteristics of the selected SNPs are presented in Table 2.

Molecular genetic testing was performed by allele-specific real-time polymerase chain reaction (real-time PCR) with fluorescently labeled TaqMan probes (Applied Biosystems, Waltham, MA, USA). Per each analyzed sample, 10 μL of reaction mixture containing 1.25 μL of appropriate TaqMan probe (Applied Biosystems, Waltham, MA, USA), 5 μL of TaqMan™ Universal PCR Master Mix (Applied Biosystems, Waltham, MA, USA), 1.75 μL of DNase-free water, and 2 μL of 100 ng genome DNA template was prepared. The amplification was performed using the ViiA 7 Real-Time PCR System (Applied Biosystems, Waltham, MA, USA) in 96-well PCR plates as follows: 10 min at 95 °C (one cycle), 15 s at 95 °C (one cycle), and 60 s at 60 °C (40 cycles). As a negative control, a reaction mixture without the genomic DNA template was used. Results of genotyping were analyzed using the QuantStudio™ Real-Time PCR Software v.1.3 (Applied Biosystems, Waltham, MA, USA). The quality of the PCR was evaluated by repeated genotyping of 10% of the samples.

### 2.3. Statistical Analysis

Statistical analysis was performed using STATISTICA 10.0 Software (StatSoft, Tulsa, OK, USA). Quantitative data were tested using the Yates’ chi-square test or the Fisher exact test. The genotyping results were analyzed using the SNPStats web tool. The most likely inheritance model for each specific gene polymorphism was determined using Akaike’s information criterion (AIC). The results are presented as the odds ratio (OR) and the 95% confidence interval (CI) calculated using five inheritance models (codominant, dominant, recessive, over-dominant, and log-additive). The differences were considered statistically significant at *p* < 0.05.

## 3. Results

Four SNPs associated with an increased risk of obesity were discovered—two variants in immune response genes (*IL6R* rs2229238, OR = 1.92, 95% CI = 1.36–2.7, *p* = 0.0002 in the dominant model; *IL18* rs1946518, OR = 1.45, 95% CI = 1.03–2.04, *p* = 0.033 in the over-dominant model) and two variants in lipid metabolism genes (*LPA* rs10455872, OR = 1.86, 95% CI = 1.07–3.21, *p* = 0.026 in the log-additive model; *LEPR* rs1137100, OR = 2.88, 95% CI = 1.52–5.46, *p* = 0.001 in the recessive model). It was inferred that the A/A genotype (recessive model) of the *TNF* gene (rs1800629) was associated with a high risk of presenting an obesity phenotype (OR = 10.29, 95% CI 1.22–86.59, *p* = 0.0081). However, reliable conclusions concerning the pathogenetic effect of this SNP could not be drawn, since this genotype was discovered in only 0.3% of nonobese patients. Moreover, the recessive models of the *CXCL8* gene (rs4073 and rs2227306) were characterized by a protective effect (OR = 0.56, 95% CI = 0.37–0.86, *p* = 0.0065 and OR = 0.49, 95% CI = 0.31–0.79, *p* = 0.0025, respectively (Table 3).

After stratification by gender, we found that the T/C genotype in the *IL6R* gene (rs2229238) and the G/G genotype in the *LEPR* gene (rs1137100) were associated with an increased risk of obesity only in males (OR = 2.27, 95% CI = 1.40–3.70, *p* = 0.0003 and OR = 2.80, 95% CI = 1.27–6.17, *p* = 0.028, respectively), while the T/G genotype in the *IL18* gene (rs1946518) was associated with an increased risk of obesity only in females (OR = 2.02, 95% CI = 1.07–3.83, *p* = 0.03). A protective effect was shown for the T/T genotype in the *CXCL8* gene (rs2227306) in females (OR = 0.44, 95% CI = 0.20–0.95, *p* = 0.04) and the G/G genotype in the *IL1RL1* gene (rs11685424) in males (OR = 0.46, 95% CI = 0.23–0.94, *p* = 0.023) (Table 4).

In the group of middle-aged patients (age ≤60 years), the G/G genotype in the *LEPR* gene (rs1137100) was associated with a fourfold increased risk of obesity (OR = 4.23, 95% CI = 1.74–10.28, *p* = 0.03). The same tendency was shown for the T allele in the *IL6R* gene (rs2229238). Among elderly patients (age >60 years), a risk association was shown for the G/A genotype in the *CRP* gene (rs1130864) (OR = 1.98, 95% CI = 1.03–3.83, *p* = 0.01) and for the C allele in the *TLR2* gene (rs3804099). Furthermore, the middle-aged patients with the A/A and T/T genotypes in the *CXCL8* gene (rs4073 and rs2227306, respectively) had a twofold decreased risk of obesity (Table 5). In contrast, the C allele in the *IL6R* gene (rs2228145) was associated with a decreased risk of obesity in middle-aged patients, whereas this allele acquired was associated with an increased risk of obesity development in elderly patients (Table 5).

## 4. Discussion

Chronic inflammation is involved in the pathogenesis of many diseases including obesity, type 2 diabetes mellitus, and atherosclerosis [15]. Danger signals caused by molecular patterns of microbial agents and endogenous damage factors (PAMPs and DAMPs) trigger the assembly of innate immunity intracellular sensors, which leads to the activation of caspase-1 and the production of proinflammatory cytokines *IL1β* and *IL18* [16]. Interleukin-18 (IL18) is an important proinflammatory cytokine involved in the pathogenesis of acute coronary events and type 2 diabetes mellitus [17], and it is associated with the modification of obesity and metabolic syndrome risk, although the underlying mechanisms remain unclear [18]. It is known that IL18R and IL18 expression in adipose tissue is enhanced in nondiabetic obesity, and it is associated with a proinflammatory gene signature and insulin resistance in such patients [19]. Polymorphic variant rs1946518 in the *IL18* gene is located in the promoter region and is associated with type 1 and 2 diabetes mellitus [20,21]. In was shown that the NLRP3 inflammasomes regulate adipose tissue metabolism via promoting IL18 secretion [22]. Despite the fact that this SNP was not associated with a metabolic syndrome in a northern Iranian population [23], we found an association of this polymorphic variant with a risk of obesity in females. We suppose that the T/G genotype of the *IL18* gene (rs1946518) is associated with increased activity of proinflammatory IL18, which interacts with the IL18Rα/β heterodimer receptor complex expressed mainly by immune cells (e.g., macrophages, dendritic cells, T and B lymphocytes), in addition to endothelial and smooth muscle cells, thus stimulating these cells in an autocrine/paracrine manner [19,24]. An increased number of these cells, especially macrophages, can be found in the expanding adipose tissue in obese patients [25].

Interleukin-6 (IL6) is a pleiotropic cytokine involved in both immune and nonimmune events in numerous cells and tissues outside of the immune system [26]. IL6 activates an intracellular signaling cascade leading to inflammation via binding to its receptor IL6R [27]. It was reported that *IL6R* gene polymorphism is associated with BMI and obesity [28,29,30]. The results herein describing an increased risk of obesity in middle-aged males carrying the C/T genotype of the *IL6R* gene (rs2229238) are consistent with the findings of an association between the C/T genotype of this gene and an increased risk of obesity in schoolboys from Taiwan [31]. We suppose that the C/T genotype is associated with an elevated serum IL6R concentration and an increased level of the IL6/IL6R complex, resulting in greater IL6 signal transduction and IL6 production, with an effect on adipocytes and immune cells in adipose tissue, as well as on insulin-targeting cells in peripheral tissues [7,31].

Lipoprotein(a), encoded by the *LPA* gene, is a serine protease with inhibition activity toward tissue plasminogen activator I. The encoded protein is proteolytically cleaved, resulting in fragments that can attach to atherosclerotic lesions and promote thrombogenesis. Elevated plasma levels of this protein are linked to atherosclerosis [32]. *LPA* genetic polymorphism is associated with different cardiovascular pathologies, e.g., coronary artery disease, aortic valve stenosis, and valvular calcification [33,34,35,36]. In the presented research, we determined for the first time an association between *LPA* gene polymorphism (rs10455872) and obesity risk according to the log-additive inheritance model, regardless of gender and age. We hypothesize that the log-additive inheritance model was characterized by some defects in the expression of lipoprotein(a) linked to apoprotein B-100, thus leading to an increase in its synthetic rate and, consequently, an elevated obesity risk.

The protein encoded by the *LEPR* gene is a receptor for leptin (an adipocyte-specific hormone regulating lipid metabolism). Mutations in this gene are associated with obesity and pituitary dysfunction. *LEPR* gene polymorphism is associated with early onset of severe obesity and hyperphagic eating behavior [37]. The G/G genotype of the *LEPR* gene (rs1137100) is potentially associated with increased expression of the leptin receptor located in hypothalamic tissue, which has a significant role in controlling energy homeostasis and lipid metabolism. The increased expression of the leptin receptor results in more active binding to leptin, whose elevated secretion by adipocytes is associated with the increased obesity risk. Our results are consistent with the literature data showing that the minor allele of the *LEPR* gene (rs1137100) is more frequent in obese patients from different populations [38,39].

An increased serum level of inflammatory markers and acute phase proteins, including C-reactive protein (CRP), is observed in obese patients [40]. It has been suggested that CRP has a direct role in the regulation of adiposity via affecting the action of adipokines [41]. Human CRP can dissociate into a physiologically active and proinflammatory monomeric form, which can bind to cell surface receptors [42] and is potentially involved in the pathogenesis of inflammatory diseases [43]. An association was revealed between CRP and leptin level [44], and a direct effect of leptin on CRP production by hepatocytes was discovered. Therefore, CRP is potentially involved in lipid metabolism via an adipo-hepato axis (leptin produced by adipocytes enhances CRP expression, which in turn may antagonize leptin action by limiting its tissue availability) [45]. Our results demonstrate that genetically determined changes in CRP production can affect the adipo-hepato axis, leading to the defects in lipid metabolism and an increased risk of obesity, but only in elderly individuals.

Defects in the relationship between adipocytes and macrophages play an important role in the initiation of adipose tissue inflammation, thereby triggering obesity [46,47,48]. Metabolic disorders lead to disbalance between pro- and anti-inflammatory regulators of macrophages toward the formation of proinflammatory M1-macrophages, which is linked to adipocyte dysfunction and the development of chronic inflammation in adipose tissue [48]. Toll-like receptors (TLRs) represent a possible pathophysiological link between obesity and inflammation. TLRs are widely represented on the surface of immune cells (macrophages, dendritic cells, neutrophils, basophils, B and T lymphocytes, natural killer cells) and nonimmune cells (fibroblasts, epithelial cells, keratinocytes) [49]. Moreover, adipocytes also express TLRs that actively participate not only in antibacterial defense, but also in the initiation of chronic inflammation of adipose tissue [50]. Enhanced lipolysis in adipocytes leads to an increase in the level of unsaturated fatty acids, which, through TLRs, promote the differentiation of macrophages into an M1 phenotype [51]. We found that the C allele of the *TLR2* gene (rs3804099) was associated with a threefold increase in risk of obesity in elderly patients due to more active TLR-promoted inflammation. 

Our most interesting results were the sex- and age-specific associations of immune response and lipid metabolism gene polymorphisms with the risk of obesity in the studied cohort. The gender dimorphism of biological and physiological functions is caused by gender-based chromosomal differences in gonadal hormone secretion. In humans, the level of gonadal hormones not only varies between males and females, but also changes depending on age, and this physiological alteration can influence the function of gonadal. hormone-sensitive genes [52]. Gonadal hormones can significantly modulate cell signaling pathways and control gene regulation and expression. It was shown that gonadal hormones may modify the immune response via regulating the production of pro- and anti-inflammatory cytokines and TLR expression [53,54,55,56]. Moreover, lipid metabolism also varies according to gender and age. Recently, a gender-specific association of *FTO* gene polymorphism with risk of obesity was revealed [57]. Therefore, the gender- and age-modulated associations of SNPs in the inflammatory response and lipid metabolism genes with obesity risk identified in the present study suggest the role of gene–gender interactions in the development of this pathology. It should be noted that our results need to be replicated in different populations with a larger sample size.

## 5. Conclusions

Genetic polymorphisms in the immune response and lipid metabolism genes are associated with increased obesity risk in middle-aged and elderly Caucasian patients in a gender- and age-depended manner. The obtained results can be used to assess the personalized risk of obesity in healthy donors during medical examination or screening, as well as to develop appropriate early prevention strategies targeting obesity in at-risk groups.

## Figures and Tables

**Table 1:** Characteristics of patients included in the study.

Index	Number (%)
Male	319 (57)
Female	241 (43)
Age ≤60 years (middle-aged patients)	382 (68)
Age >60 years (elderly patients)	178 (32)
BMI ≥30 kg/m^2^	220 (39)
BMI ≥30 kg/m^2^ in middle-aged patients	156 (41)
BMI ≥30 kg/m^2^ in elderly patients	64 (36)

**Table 2:** Characteristics of the studied polymorphic variants.

Gene	Reference SNP Number	Chromosomal Position	Nucleotide Change	Variant Type
*TLR1*	rs5743611	chr4:38798593	C > G	Missense variant
	rs5743551	chr4:38806033	T > A, C, G	5’ UTR variant
*TLR2*	rs5743708	chr4:153705165	G > A	Missense variant
*TLR4*	rs4986791	chr9:117713324	C > T	Missense variant
	rs4986790	chr9:117713024	A > G, T	Missense variant
*TLR6*	rs5743810	chr4:38828729	A > C, G, T	Missense variant
	rs3775073	chr4:38828211	T > C, G	Missense variant
*IL1RL1*	rs4988956	chr2:102351547	G > A	Missense variant
	rs11685424	chr2:102310521	G > A	Upstream transcript variant
*IL1B*	rs1143634	chr2:112832813	G > A	Synonymous variant
	rs16944	2:112837290	A > G	Upstream transcript variant
*IL6R*	rs2228145	chr1:154454494	A > C, T	Missense variant
	rs2229238	chr1:154465420	T > A, C	3’ UTR variant
*IL6*	rs1800796	chr7:22726627	G > A, C	Intron variant
	rs1554606	chr7:22729088	T > A, G	Intron variant
	rs2069827	chr7:22725837	G > C, T	Upstream transcript variant
*CXCL8*	rs2227306	chr4:73741338	C > T	Intron variant
	rs4073	chr4:73740307	A > C, G, T	Upstream transcript variant
*IL10*	rs1800871	chr1:206773289	A > G	Upstream transcript variant
	rs1800872	chr1:206773062	T > G	Upstream transcript variant
	rs1800896	chr1:206773552	T > C	Upstream transcript variant
*IL12RB1*	rs375947	chr19:18069641	A > G	Missense variant
*IL12B*	rs3212227	chr5:159315942	T > G	3’ UTR variant
*IL18RAP*	rs917997	chr2:102454108	T > A, C, G	Not announced
	rs2058659	chr2:102438096	G > A	Intron variant
*IL18R1*	rs13015714	chr2:102355405	G > A, T	Upstream transcript variant
	rs1974675	chr2:102369915	G > A	Intron variant
	rs6758936	chr2:102374909	G > A	Intron variant
	rs3755276	chr2:102361999	C > T	Intron variant
*IL18*	rs187238	chr11:112164265	C > A, G	Upstream transcript variant
	rs360719	chr11:112165426	A > G	Upstream transcript variant
	rs1946518	chr11:112164735	T > G	Upstream transcript variant
*IL33*	rs7025417	chr9:6240084	T > C, G	Intron variant
*TNF*	rs1799964	chr6:31574531	T > C	Upstream transcript variant
	rs361525	chr6:31575324	G > A	Upstream transcript variant
	rs1800629	chr6:31575254	G > A	Upstream transcript variant
*CRP*	rs3093077	chr1:159709846	A > C, G, T	Not announced
	rs1800947	chr1:159713648	C > A, G, T	Synonymous variant
	rs1130864	chr1:159713301	G > A	Intron variant
	rs1205	chr1:159712443	C > T	3’ UTR variant
*APOE*	rs429358	chr19: 44908684	T > C	Missense variant
	rs769452	chr19:44907853	T > A, C	Missense variant
	rs7412	chr19:44908822	C > T	Missense variant
*APOB*	rs1042031	chr2:21002881	C > A, T	Missense variant/Stop gained
	rs6725189	chr2:20996129	G > T	Not announced
*LPA*	rs10455872	chr6:160589086	A > G	Intron variant
*LIPC*	rs1800588	chr15:58431476	C > G, T	Intron variant
*CXCR1*	rs16858811	chr2:218165120	A > C	Missense variant
*CXCR2*	rs1126579	chr2:218136011	T > C	3’ UTR variant
*INS*	rs689	chr11:2160994	A > G, T	Intron variant
*IGF1R*	rs2229765	chr15:98934996	G > A, T	Missense variant
*LEP*	rs7799039	chr7:128238730	G > A, C	Not announced
*LEPR*	rs1137101	chr1:65592830	A > G, T	Missense variant
	rs1137100	chr1:65570758	A > G, T	Missense variant
*IL1F9*	rs17659543	chr2:112958729	C > T	Not announced

**Table 3:** Association of SNPs with risk of obesity, adjusted by gender and age.

Gene	Model	Genotype	No Obesity, *N* (%)	Obesity, *N* (%)	OR (95% CI)	*p*	AIC
*IL6R* rs2229238	Codominant	C/C	189 (55.9)	88 (40)	1.00	0.0009	741.5
		T/C	123 (36.4)	112 (50.9)	1.97 (1.37–2.83)		
		T/T	26 (7.7)	20 (9.1)	1.66 (0.88–3.14)		
	Dominant	C/C	189 (55.9)	88 (40)	1.00	0.0002	739.8
		T/C-T/T	149 (44.1)	132 (60)	**1.92 (1.36–2.71)**		
	Recessive	C/C-T/C	312 (92.3)	200 (90.9)	1.00	0.56	753.3
		T/T	26 (7.7)	20 (9.1)	1.20 (0.65–2.21)		
	Over-dominant	C/C-T/T	215 (63.6)	108 (49.1)	1.00	0.0006	741.9
		T/C	123 (36.4)	112 (50.9)	1.83 (1.29–2.58)		
	Log-additive	-	-	-	1.53 (1.17–2.01)	0.0016	743.7
*CXCL8* rs4073	Codominant	T/T	91 (26.8)	71 (32.3)	1.00	0.022	748.8
		A/T	154 (45.4)	109 (49.5)	0.90 (0.61–1.34)		
		A/A	94 (27.7)	40 (18.2)	0.53 (0.33–0.86)		
	Dominant	T/T	91 (26.8)	71 (32.3)	1.00	0.15	752.4
		A/T-A/A	248 (73.2)	149 (67.7)	0.76 (0.53–1.11)		
	Recessive	T/T-A/T	245 (72.3)	180 (81.8)	1.00	0.0065	747
		A/A	94 (27.7)	40 (18.2)	**0.56 (0.37–0.86)**		
	Over-dominant	T/T-A/A	185 (54.6)	111 (50.5)	1.00	0.32	753.5
		A/T	154 (45.4)	109 (49.5)	1.19 (0.85–1.67)		
	Log-additive	-	-	-	0.74 (0.58–0.94)	0.013	748.3
*CXCL8* rs2227306	Codominant	C/C	103 (30.3)	77 (35)	1.00	0.0098	748
		C/T	160 (47.1)	115 (52.3)	0.94 (0.64–1.38)		
		T/T	77 (22.6)	28 (12.7)	0.48 (0.28–0.81)		
	Dominant	C/C	103 (30.3)	77 (35)	1.00	0.21	753.7
		C/T-T/T	237 (69.7)	143 (65)	0.79 (0.55–1.14)		
	Recessive	C/C-C/T	263 (77.3)	192 (87.3)	1.00	0.0025	746.1
		T/T	77 (22.6)	28 (12.7)	**0.49 (0.31–0.79)**		
	Over-dominant	C/C-T/T	180 (52.9)	105 (47.7)	1.00	0.25	754
		C/T	160 (47.1)	115 (52.3)	1.22 (0.87–1.71)		
	Log-additive	-	-	-	0.73 (0.57–0.94)	0.012	749
*TNF* rs1800629	Codominant	G/G	261 (77)	167 (75.9)	1.00	0.028	749.2
		G/A	77 (22.7)	47 (21.4)	0.93 (0.62–1.41)		
		A/A	1 (0.3)	6 (2.7)	10.14 (1.20–85.48)		
	Dominant	G/G	261 (77)	167 (75.9)	1.00	0.83	754.3
		G/A-A/A	78 (23)	53 (24.1)	1.05 (0.70–1.56)		
	Recessive	G/G-G/A	338 (99.7)	214 (97.3)	1.00	0.0081	747.3
		A/A	1 (0.3)	6 (2.7)	**10.29 (1.22–86.59)**		
	Over-dominant	G/G-A/A	262 (77.3)	173 (78.6)	1.00	0.63	754.1
		G/A	77 (22.7)	47 (21.4)	0.90 (0.60–1.37)		
	Log-additive	-	-	-	1.17 (0.81–1.69)	0.41	753.6
*IL18* rs1946518	Codominant	G/G	111 (32.6)	63 (28.9)	1.00	0.066	748.3
		T/G	158 (46.5)	122 (56)	1.32 (0.89–1.95)		
		T/T	71 (20.9)	33 (15.1)	0.78 (0.46–1.31)		
	Dominant	G/G	111 (32.6)	63 (28.9)	1.00	0.45	751.2
		T/G-T/T	229 (67.3)	155 (71.1)	1.15 (0.79–1.68)		
	Recessive	G/G-T/G	269 (79.1)	185 (84.9)	1.00	0.062	748.3
		T/T	71 (20.9)	33 (15.1)	0.65 (0.41–1.03)		
	Over-dominant	G/G-T/T	182 (53.5)	96 (44)	1.00	0.033	747.2
		T/G	158 (46.5)	122 (56)	**1.45 (1.03–2.04)**		
	Log-additive	-	-	-	0.93 (0.73–1.20)	0.59	751.5
*LPA* rs10455872	Codominant	A/A	315 (92.7)	191 (87.6)	1.00	0.032	746.6
		A/G	25 (7.3)	25 (11.5)	1.64 (0.92–2.95)		
		G/G	0 (0)	2 (0.9)	NA (0.00-NA)		
	Dominant	A/A	315 (92.7)	191 (87.6)	1.00	0.049	747.6
		A/G-G/G	25 (7.3)	27 (12.4)	1.79 (1.00–3.17)		
	Recessive	A/A-A/G	340 (100)	216 (99.1)	1.00	0.042	747.3
		G/G	0 (0)	2 (0.9)	N/A (0.00-N/A)		
	Over-dominant	A/A-G/G	315 (92.7)	193 (88.5)	1.00	0.1	748.8
		A/G	25 (7.3)	25 (11.5)	1.62 (0.91–2.92)		
	Log-additive	-	-	-	**1.86 (1.07–3.21)**	0.026	746.5
*LEPR* rs1137100	Codominant	A/A	181 (53.4)	97 (44.5)	1.00	0.0021	740.4
		A/G	141 (41.6)	94 (43.1)	1.24 (0.87–1.78)		
		G/G	17 (5)	27 (12.4)	3.19 (1.64–6.18)		
	Dominant	A/A	181 (53.4)	97 (44.5)	1.00	0.036	746.2
		A/G-G/G	158 (46.6)	121 (55.5)	1.44 (1.02–2.03)		
	Recessive	A/A-A/G	322 (95)	191 (87.6)	1.00	0.001	739.8
		G/G	17 (5)	27 (12.4)	**2.88 (1.52–5.46)**		
	Over-dominant	A/A-G/G	198 (58.4)	124 (56.9)	1.00	0.76	750.6
		A/G	141 (41.6)	94 (43.1)	1.06 (0.75–1.49)		
	Log-additive	-	-	-	1.53 (1.16–2.00)	0.0021	741.2

**Note:** Statistically significant results after applying Akaike’s information criterion (AIC) are highlighted in bold.

**Table 4:** Association of SNPs with risk of obesity in groups stratified by gender.

Gene	Gender	Genotype	No Obesity, *N*	Obesity, *N*	OR (95%CI)	*p*
*IL6R* rs2229238	Male	C/C	112	43	1.00	0.002
		T/C	73	64	**2.27 (1.40–3.70)**	
		T/T	15	10	1.74 (0.73–4.17)	
	Female	C/C	77	45	1.00	0.08
		T/C	50	48	1.65 (0.96–2.83)	
		T/T	11	10	1.57 (0.62–4.00)	
*CXCL8* rs2227306	Male	C/C	66	42	1.00	0.5
		C/T	92	61	1.04 (0.63–1.72)	
		T/T	44	14	0.51 (0.25–1.04)	
	Female	C/C	37	35	1.00	0.04
		C/T	68	54	0.83 (0.46–1.49)	
		T/T	33	14	**0.44 (0.20–0.95)**	
*IL1RL1* rs11685424	Male	A/A	58	37	1.00	0.023
		G/A	91	65	1.12 (0.67–1.89)	
		G/G	51	15	**0.46 (0.23–0.94)**	
	Female	A/A	45	30	1.00	0.43
		G/A	67	49	1.11 (0.61–2.01)	
		G/G	26	24	1.39 (0.68–2.87)	
*IL18* rs1946518	Male	G/G	70	43	1.00	0.48
		T/G	94	59	1.01 (0.61–1.66)	
		T/T	38	15	0.63 (0.31–1.28)	
	Female	G/G	41	20	1.00	0.03
		T/G	64	63	**2.02 (1.07–3.83)**	
		T/T	33	18	1.10 (0.50–2.41)	
*LEPR* rs1137100	Male	A/A	105	53	1.00	0.028
		A/G	83	45	1.07 (0.66–1.75)	
		G/G	13	18	**2.80 (1.27–6.17)**	
	Female	A/A	76	44	1.00	0.004
		A/G	58	49	1.48 (0.87–2.53)	
		G/G	4	9	4.04 (1.17–13.94)	

**Note:** Statistically significant results are highlighted in bold.

**Table 5:** Association of SNPs with obesity risk in groups stratified by age.

Gene	Age	Genotype	No Obesity, *N*	Obesity, *N*	OR (95%CI)	*p*
*IL6R* rs22281454	≤60 years	A/A	94	88	1.00	0.004
		A/C	107	59	**0.58 (0.38–0.90)**	
		C/C	24	9	**0.40 (0.18–0.90)**	
	>60 years	A/A	63	25	1.00	0.027
		A/C	40	32	**2.03 (1.05–3.91)**	
		C/C	10	7	1.70 (0.58–4.98)	
*IL6R* rs2229238	≤60 years	C/C	130	59	1.00	0.015
		T/C	82	81	**2.21 (1.43–3.41)**	
		T/T	14	16	**2.53 (1.16–5.53)**	
	>60 years	C/C	59	29	1.00	0.49
		T/C	41	31	1.54 (0.80–2.93)	
		T/T	12	4	0.67 (0.20–2.25)	
*CXCL8* rs4073	≤60 years	T/T	59	50	1.00	0.027
		A/T	100	79	0.93 (0.58–1.51)	
		A/A	67	27	**0.46 (0.26–0.83)**	
	>60 years	T/T	32	21	1.00	0.07
		A/T	54	30	0.84 (0.41–1.71)	
		A/A	27	13	0.74 (0.31–1.75)	
*CXCL8* rs2227306	≤60 years	C/C	65	54	1.00	0.04
		C/T	110	82	0.89 (0.56–1.40)	
		T/T	51	20	**0.45 (0.24–0.86)**	
	>60 years	C/C	38	23	1.00	0.05
		C/T	50	33	1.08 (0.55–2.14)	
		T/T	26	8	0.52 (0.20–1.36)	
*CRP* rs1130864	≤60 years	G/G	115	79	1.00	0.19
		G/A	98	62	0.91 (0.59–1.39)	
		A/A	13	15	1.70 (0.77–3.78)	
	>60 years	G/G	55	22	1.00	0.01
		G/A	47	37	**1.98 (1.03–3.83)**	
		A/A	12	5	1.04 (0.33–3.29)	
*LEPR* rs1137100	≤60 years	A/A	125	70	1.00	0,03
		A/G	93	67	1.29 (0.84–1.98)	
		G/G	8	18	**4.23 (1.74–10.28)**	
	>60 years	A/A	56	27	1.00	0.15
		A/G	48	27	1.14 (0.59–2.20)	
		G/G	9	9	2.12 (0.75–5.98)	
*TLR2* rs3804099	≤60 years	T/T	86	64	1.00	0.36
		T/C	106	68	0.86 (0.55–1.34)	
		C/C	34	24	0.95 (0.52–1.77)	
	>60 years	T/T	56	16	1.00	0.01
		T/C	41	30	**2.55 (1.23–5.28)**	
		C/C	17	17	**3.35 (1.39–8.04)**	

**Note:** Statistically significant results are highlighted in bold.

## Data Availability

The data presented in this study are available on request from the corresponding author. The data are not publicly available due to privacy statements.
